# Ectopic overexpression of Kir6.1 in the mouse heart impacts on the life expectancy

**DOI:** 10.1038/s41598-018-30175-5

**Published:** 2018-08-06

**Authors:** Yasuhiro Watanabe, Takashi Kishimoto, Takashi Miki, Susumu Seino, Haruaki Nakaya, Akio Matsumoto

**Affiliations:** 1 Department of Pharmacology, Graduate School of Medicine, Chiba University, Chiba, Japan; 2 Department of Molecular Pathology, Graduate School of Medicine, Chiba University, Chiba, Japan; 3 Department of Medical Physiology, Graduate School of Medicine, Chiba University, Chiba, Japan; 4 Division of Molecular and Metabolic Medicine, Kobe University Graduate School of Medicine, Kobe, Japan

## Abstract

We recently reported the reduced ATP-sensitive potassium (K_ATP_) channel activities in the transgenic mouse heart overexpressing the vascular type K_ATP_ channel pore-forming subunit (Kir6.1). Although dysfunction of cardiac K_ATP_ channel has been nominated as a cause of cardiomyopathy in human, these transgenic mice looked normal as wild-type (WT) during the experiment period (~20 weeks). Extended observation period revealed unexpected deaths beginning from 30 weeks and about 50% of the transgenic mice died by 55 weeks. Surface ECG recordings from the transgenic mice at rest demonstrated the normal sinus rhythm and the regular ECG complex as well as the control WT mice except for prolonged QT interval. However, the stress ECG test with noradrenaline revealed abnormal intraventricular conduction delay and arrhythmogeneity in the transgenic mouse. Fibrotic changes in the heart tissue were remarkable in aged transgenic mice, and the cardiac fibrosis developed progressively at least from the age of 30 weeks. Gene expression analyses revealed the differentiation of cardiac fibroblasts to myofibroblasts with elevated cytokine expressions was initiated way in advance before the fibrotic changes and the upregulation of BNP in the ventricle. In sum, Kir6.1TG mice provide an electro-pathological disease concept originated from K_ATP_ channel dysfunction.

## Introduction

K_ATP_ channel (ATP-sensitive potassium channel) is a metabolic sensor channel which reflects changes in cell metabolism (ATP) into the electrophysiological membrane potential through the modulation of potassium ion flux^1^. Although the K_ATP_ channel remains close-state under physiological circumstances in cardiomyocytes, the anoxic conditions such as coronary occlusion cause transition of the cardiac K_ATP_ channel to the open-state due to the depletion of intracellular ATP. Thus, the K_ATP_ channel plays a cardioprotective role by shortening the action potential duration, which then inhibits the excessive influx and accumulation of Ca^2+^ in cardiomyocytes^2–4^.

The K_ATP_ channel is a hetero-octameric protein of four pore-forming Kir6 and four sulfonylurea receptor (SUR) subunits^1,5^. The sarcolemmal K_ATP_ channels in ventricular myocytes are composed primarily of Kir6.2 (also known as *KCNJ11*) and SUR2A (also known as *ABCC9*) subunits whereas K_ATP_ channels in vascular smooth muscle cells are predominantly composed of Kir6.1 (also known as *KCNJ8*) and SUR2B (a splice variant of SUR2A)^1,2^.

Dysfunction of the sarcolemmal K_ATP_ channel has been proposed as a causative factor of the heart failure and various cardiovascular diseases based on the accumulated evidence from the mouse studies^2,6–9^, and human cases^10^. A genetic defect in the K_ATP_ channel components, such as Kir6.1, Kir6.2, and SUR2, has brought the detrimental consequences in the mouse heart. For example, genetic disruption of mouse Kir6.1 gene causes hypercontractility of coronary arteries resembling Prinzmetal angina in humans^6^. Kir6.2-null mice do not have cardiac K_ATP_ channel current^2^, show a maladaptive phenotype with increased vulnerability under stress^3,11^. Kir6.2-null mice fail to adapt the stress conditions through sympathetic stimulation since catecholamine challenge caused ventricular arrhythmia and death. In human hereditary genetic mutation has been pointed out as a cause of dilated cardiomyopathy and ischemic cardiomyopathy due to the loss of function of the K_ATP_ channel in cardiomyocytes^10,12^. These findings established a disease concept of ion-channel related cardiac dysfunction, cardiac channelopathy^4^.

We recently reported the downregulation of K_ATP_ channel in cardiomyocyte from the transgenic mouse overexpressing vascular form of K_ATP_ channel pore-forming subunit Kir6.1 driven by the α-MHC promoter^13^. The transgenic mouse strains (termed as Kir6.1TG) showed the prolonged QT interval, which was supported by the extended action potential duration (APD) by electrophysiological analyses with the patch-clamp techniques. Further studies revealed that K_ATP_ current (*I*_KATP_) densities were significantly lower in Kir6.1TG than wild-type (WT) controls, as well as the voltage-dependent potassium currents such as *I*_to_, *I*_K_, and *I*_K1_. This evidence might be explained as the dominant-negative effect of overexpressed Kir6.1 in the cardiac K_ATP_ channel in which Kir6.2 and SUR2A form a channel subunit^5^. Although the K_ATP_ channel activity is suppressed in the heart, there were no noticeable differences in the growth and survival between Kir6.1TG and WT mice during the experiment period (until 20 weeks after birth). The evidence that the K_ATP_ channel in the Kir6.1TG mouse heart does not function like WT led us to conduct the further investigation with extended observation period for more than a year to see the life expectancy of the Kir6.1TG mice.

## Results

### Life expectancy of Kir6.1TG mouse strains

We reported previously that Kir6.1TG mice exhibit atypical patterns in non-stress ECG recordings^13^. Distinct changes were either the depression of the J-ST segment or the enlarged J wave. Extended QT/QTc intervals were seen in all Kir6.1TG strains. Although electrophysiological mechanisms causing J-ST segment changes need to employ further investigation, prolonged QT interval is supported by the lower density of sarcoplasmic K_ATP_ currents (*I*_KATP_), besides *I*_to_, *I*_K_, and *I*_K1_. As we limited the observation period of Kir6.1TG mice to 20 weeks from the birth in the previous experiments, there was no apparent phenotype presented by these Kir6.1TG mice compared with non-transgenic WT mice. There were no significant differences between Kir6.1TG mice and the parental strain (C57BL/6) in the growth, the reproduction rate, and the litter size. However, unexpected deaths of the retired Kir6.1TG mice elicited the possibility of the shorter lifespan of Kir6.1TG mice. Those mice were found dead within a day of displaying normal activities. Thus, we extended the observation period over a year to characterize Kir6.1TG mice.

**Figure 1:**
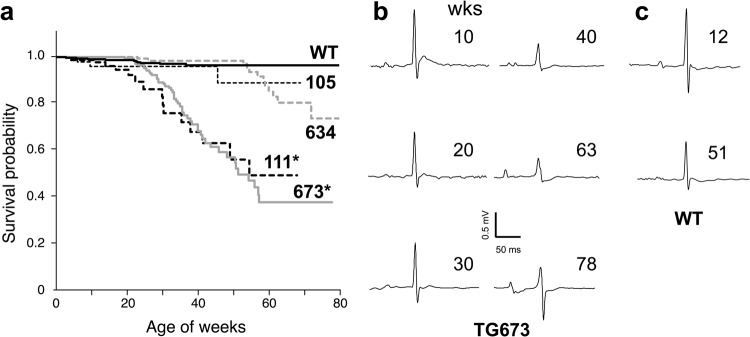
Overall survival among Kir6.1TG mice and changes in ECG traces. (**a**) Kaplan-Meier curves for overall survival among four Kir6.1TG lines. Line 673 and 634 expresses wild-type Kir6.1, while line 111 and 105 expresses mutant Kir6.1 (S422L). These lines derived from the distinct founders. Each curve enrolled the following number of mice, WT:1737, 634:247, 673:327, 105:50, 111:87, respectively. *p < 0.05 vs. WT. (**b**,**c**) Representative ECG traces from Kir6.1TG (line 673) mice (**b**) and WT control (**c**) at different stages of life. Surface ECG (lead-II) was recorded from the anesthetized mouse.

As life expectancy showed by the Kaplan-Meier curve in Fig. 1a, deaths in Kir6.1TG mice accumulated over time, although the number of mice found dead in the cage was very limited in the parental WT mice. These observed deaths were unpredictable as the mouse stayed as usual on one day and found dead on the next day, suggesting the sudden death of cardiac origin. The Kir6.1TG consist of two independent strains; one expresses wild-type Kir6.1 from the transgene (line 673 and line 634), and the other has a mutation (S422L) on Kir6.1 (line 105 and line 111). As we reported previously^13^, all four Kir6.1TG lines exhibited markedly prolonged action potential duration (APD) than WT control, supported by the fact that ventricular myocytes from Kir6.1TG mice had less K^+^ current densities than WT control. Among the four founder lines of Kir6.1TG, ventricular myocytes from lines 673 and 111 show relatively larger *I*_to_ and *I*_K1_ densities and somewhat smaller *I*_KATP_ densities than other lines (634 and 105), although there were no significant differences in these currents among the four Kir6.1TG lines. Kir6.1TG mice in lines 673 and 111 died significantly earlier with the median survival between 52 to 55 weeks of age than non-transgenic WT control (Fig. 1a; more than 95% of WT mice survived longer than 80 weeks). Although lines 673 and 111 harboring WT or S422L mutant of Kir6.1, respectively, survival curves of these lines were almost the same. Further, line 634 survived longer than lines 673 and 111; however, the curve starts descending at the similar rate in their later life. These results suggest that the overexpression of Kir6.1 in cardiomyocytes is a cause of death in these mice. There was no sex difference in the life expectancy (Supplementary Fig. S1). Thus, we focused on line 673 as a representative of the Kir6.1TG mouse.

As these Kir6.1TG mice show atypical ECG changes from their early life, surface ECG recordings (lead II) were compared between individuals at different ages (line 673 from 10 to 78 weeks) as shown in Fig. 1b. These traces exhibited significant decreases of the R-wave amplitude and the depression in the ST-segment particularly after 40 weeks, which indicate the electrophysiological suppression in the heart, particularly in cardiomyocytes, although the R-wave amplitude in aged WT controls was slightly lower than the younger age (Fig. 1c).

### Pharmacologic stress ECG test

It was difficult to define the incidence of these Kir6.1TG mice since the disease progress was gradual and the death happened without a sign. It was proposed that the sudden death may be triggered in combination with other factors, such as sympathetic stimulations. A suggestive case was a male Kir6.1TG mouse found dead on the next day following mating with a female mouse. There was no finding of the indicative cause of death on the mouse, and it was too late to do the pathological examination. Thus, pharmacologic stress test with noradrenaline as a cardiovascular stressor was employed to investigate the cardiac sensitivity and durability to sympathetic stimulation as a cause of unexpected deaths found in Kir6.1TG mice.

**Figure 2:**
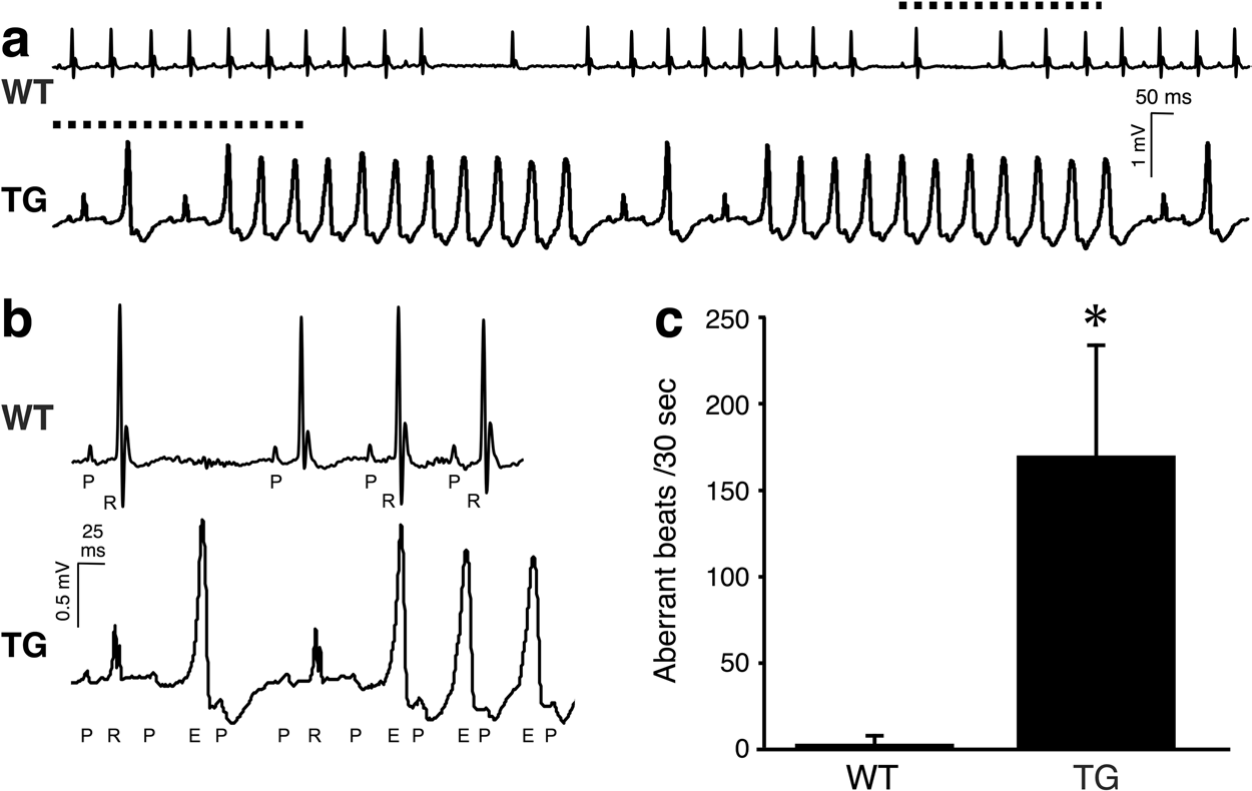
Atypical ECG patterns observed in the stress-ECG recordings. Surface ECG (lead-II) was recorded from anesthetized mouse over 40 weeks of age. (**a**) An administration of noradrenaline (1 mg/kg.BW i.p.) induced sinus tachycardia in WT and Kir6.1TG (line 673). WT strain occasionally showed missing regular contraction. Kir6.1TG strain frequently showed a series of aberrant conduction. The dotted line indicates the portion displayed in b. (**b**) Magnified ECG traces. WT maintains regular complex with P-wave (P) and a ventricular contraction (R). Kir6.1TG (line 673) maintains the regular rhythm of P-wave, while QRS complexes were frequently replaced with an ectopic form (E), suggesting ventricular conduction delay. The normal QRS complex of Kir6.1TG mouse also indicated fusion of the aberrant conduction with the normal conduction. (**c**) Highest occurrence of aberrant beats in a 30-sec period was analyzed between 10 and 20 minutes after noradrenaline injection. WT: n = 8, Kir6.1TG: n = 6. *p = 0.0082 (Kruskal-Wallis test).

Mice aged over 40 weeks were chosen for this stress test. Before an administration of noradrenaline, ECG showed sinus rhythm in Kir6.1TG as well as WT controls (Supplementary Fig. S2). Heart rate was in the range of 200~300/min, due to the application of medetomidine (α_2_ stimulant) as a component of anesthetics. Following an intraperitoneal administration of noradrenaline at 1 mg/kg.BW, heart rate reached to the range of 600~800/min. Heart rate response to the drug was slightly weaker in Kir6.1TG mice, but there were no significant differences observed. During the 45 minutes observation period following injection of noradrenaline, Kir6.1TG mice exhibited sustained morphological changes in ECG waveforms, although ECGs from WT mice occasionally showed missing regular heart contractions (Fig. 2a). Frequently observed changes in Kir6.1TG mice included wide-QRS complex or notched-QRS, those suggest the formation of intraventricular conduction delay or alternative conduction pathways. The morphological ECG changes recognized in Kir6.1TG mice were not consistent in all mice, there were some variations in the type of arrhythmia (Supplementary Fig. S3). One should be noted that regular atrial rhythm was maintained during the observation period in Kir6.1TG mice although some conduction failures were observed between atrium and ventricle (Fig. 2b). There were no cases that present ventricular tachycardia or fibrillation in both WT and Kir6.1TG during the observation period.

The highest counts of aberrant beats observed in 30-second were measured from 10 to 20 minutes after an administration of noradrenaline. Noradrenaline significantly induced atypical ECG patterns in Kir6.1TG mice than WT (Fig. 2c). Following the first observation period for thirty minutes, the mouse received noradrenaline at the same dose every 30 minutes at most five times. Although WT mice repeatedly responded to the drug till the end of the experiment, Kir6.1TG mice could not tolerate repeated administration of noradrenaline, and all mice died before or after the second injection. These results suggested that Kir6.1TG mouse hearts tend to induce cardiac death with abnormal ventricular conduction in response to the catecholamine stimulation.

### Pathological analyses

**Figure 3:**
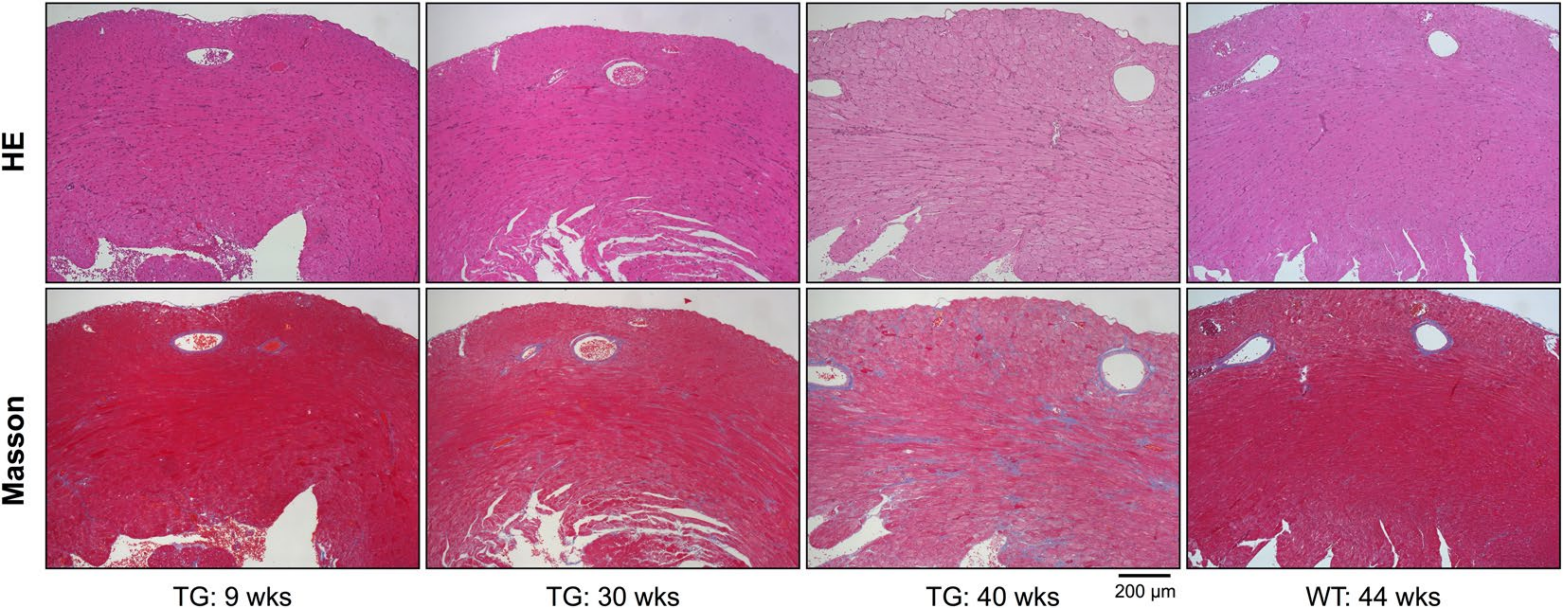
Fibrotic changes in Kir6.1TG mouse heart. Representative Hematoxylin and Eosin staining and Masson Trichrome staining of the anterior wall of the left ventricle section from WT and Kir6.1TG (line 673) mice (n = 2–3 mice per group). Aged Kir6.1TG (40 weeks) heart increases in fibrosis compared to the WT (44 weeks), and younger Kir6.1TG (9 and 30 weeks) hearts. Scale bar: 200 µm, original magnification × 100.

ECG analyses revealed that Kir6.1TG mice are catecholamine sensitive and arrhythmogenic. Microscopic studies were carried out with heart tissues to investigate the detail of electrophysiological changes. As the stress ECG recordings with mice over 40 weeks of age revealed cardiac conduction disturbance (Fig. 2), mice at 40 and 44 weeks were taken for the pathological analysis. The characteristic finding was advanced tissue fibrosis in the left ventricle of the old Kir6.1TG heart (40 weeks) (Fig. 3). WT hearts at the comparable age (44 weeks) also presented fibrotic changes with the substantially lesser degree, which could be referred to as a senile change. The fibrotic region was evenly distributed throughout the wall from endocardium to epicardium. The coronary arteries and associated microvasculature remained intact in the evaluation with EVG (Elastica van Gieson) stain. There was no apparent disarray in the myocardium; however, hypertrophic changes in myocardial cells were seen in endocardium to some extent. There were no hemosiderin-laden macrophages in the lung in Prussian blue staining, the results indicating that congestive heart failure did not occur. Because of the limited availability of the aged Kir6.1TG mice due to the increased rate of death, we could take only two Kir6.1TG mice at 40 weeks or higher for the pathological analysis. The characteristic findings from these two mice were almost identical. Further investigation was performed with mice at younger ages (9 and 30 weeks). Judging from the intensity of Masson’s trichrome staining, cardiac fibrosis was definite at 30 weeks although the distribution is mainly from the endocardium to the midcardium. The positive stain was observed only in the perivascular area within the normal range in the endocardium of Kir6.1TG heart at nine weeks. These observations indicate the progression of fibrosis with age in the Kir6.1TG mouse heart. As to support the pathological findings above from the line 673, two mice from the line 111 (with S422L mutation, the life expectancy is almost identical to the line 673, shown in Fig. 1 and Supplementary Fig. S1) were taken for the pathological analyses at 35 weeks of age and compared with the age-matched WT littermates. The fibrotic changes and the hypertrophic changes in myocardial cells were distinctive in TG mice than WT (Supplementary Fig. S4). These pathological findings were in-line with those from the line 673.

### Gene expression profiling

**Figure 4:**
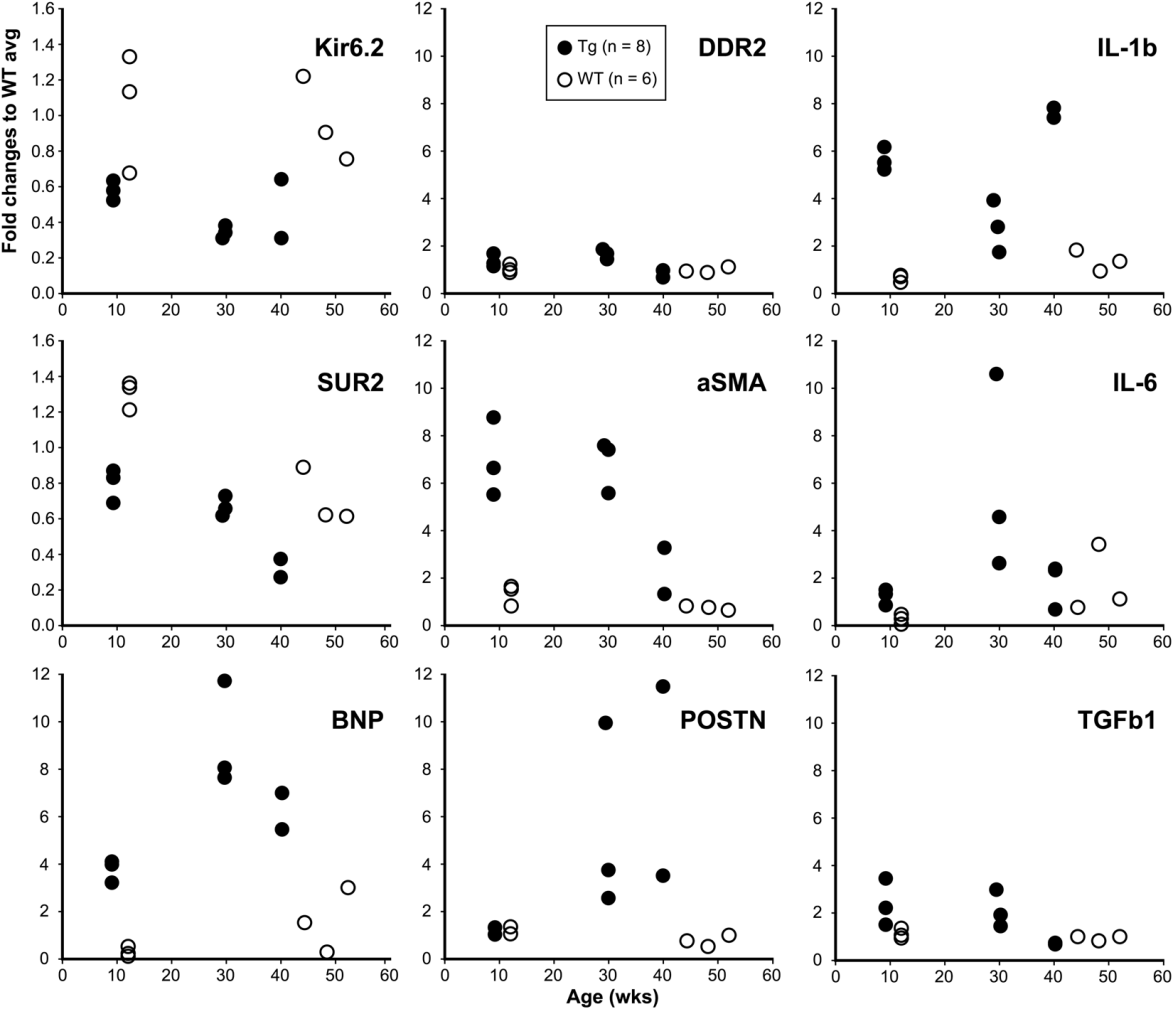
Gene expression analyses in the apex tissue by semi-quantitative real-time PCR. cDNA was synthesized from the total RNA purified from the apex of the left ventricle. Kir6.1TG mice (line 673) were sub-grouped according to the age of weeks (9, 30, 40, respectively), and compared with WT (average 48.2 weeks and 12 weeks). qPCR values were first normalized to RPL4, then calculated fold changes to the average of WT. Each circle indicates the value of each mouse (WT: n = 6, Kir6.1TG 9 weeks: n = 3, 30 weeks: n = 3, 40 weeks: n = 2). Kir6.1r: rat Kir6.1 (transgene), Kir6.2: mouse Kir6.2, SUR2: sulfonylurea receptor type 2, BNP: B-type natriuretic peptide, POSTN: periostin, DDR2: discoidin domain receptor 2, aSMA: alpha-smooth muscle actin, IL-1b: interleukine-1β, IL-6: interleukine-6, TGFb1: transforming growth factor β1.

The apex of the heart was reserved for the gene expression analysis before the fixation for the pathological examination as reported above (line 673). Following synthesis of cDNA from the heart tissue, relative gene expression levels were assayed by semi-quantitative real-time PCR (qPCR) method and summarized in Table 1 (upper row). In each group (9, 30, and 40 weeks of age) Kir6.1TG littermate mice were collected in the sample. Due to the limited availability of the aged Kir6.1TG mice (40 weeks), it was not possible to compare the difference between age-groups. Table 1 summarized the overall comparison between Kir6.1TG and WT mice (the average of WTs as 1.0). The circles in the graph plotted individual values. Since Kir6.1TG mouse has two different expression sources for Kir6.1, one from the transgene with rat Kir6.1 (termed as Kir6.1r) and the other from the endogenous mouse Kir6.1^2^, specific primer sets (summarized in Table 2) were applied to segregate the expression levels of each Kir6.1. The value for Kir6.1r in Table 1 is based on the direct comparison between mouse Kir6.1 in WT and rat Kir6.1 in the TG mouse. There was no significant difference in mouse Kir6.1 between WT and Kir6.1TG mice. Remaining values for nine genes, SUR2, Kir6.2, BNP (B-type natriuretic peptide), POSTN (Periostin), DDR2 (Discoidin Domain Receptor 2), aSMA (alpha-Smooth Muscle Actin), IL-1β (Interleukin-1β), IL-6, and TGFβ1 (Transforming Growth Factor β1) are the fold increase in expression level of Kir6.1TG mice on WT. Although there were no significant differences on SUR2 expression levels between Kir6.1TG and WT, expression levels of SUR2 in aged Kir6.1TG mice (40 weeks) were considerably lower than WT and other Kir6.1TG mice at the younger age (9 and 30 weeks) (Fig. 4 left). The similar pattern was observed in β1-adrenergic receptor expressions (Supplementary Fig. S5). BNP and POSTN were employed as a sensitive marker for heart failure^14^ and fibrosis^15^, respectively. Increased BNP levels in Kir6.1TG mice indicate the occurrence of contraction dysfunction, although the apparent pathological findings suggest that heart failure has not been developed in the lung tissue. Periostin levels were significantly higher in the group of Kir6.1TG mice (30 and 40 weeks) compared with the group including all WT and Kir6.1TG at nine weeks (p < 0.0027) in line with the pathological findings.

**Table 1:** Gene expression levels in Kir6.1TG heart tissue normalized to WT.

	Kir6.1r	Kir6.2	SUR2	BNP	POSTN	DDR2	aSMA	IL-1b	IL-6	TGFb1
TG/WT	124	0.5	0.6	6.4	4.4	1.3	5.8	5.1	3.0	1.9
	117	0.5	0.6	5.8	4.6	1.2	5.8	4.3	3.4	1.7
p value	0.002	0.002	N.S.	0.002	0.014	N.S.	0.005	0.003	N.S.	N.S.
	0.002	0.004	N.S.	0.001	0.014	N.S.	0.002	0.004	0.021	N.S.

Summarizes the fold changes of Kir6.1TG (includes all ages, upper row: n = 8 (line 673), lower row: n = 11 including line 111 with line 673) to WT. p values from Kruskal-Wallis test. N.S.: Not Significant (p > 0.05).

**Table 2:** Primer sets used for real-time PCR analyses.

Target gene	Forward 5′–3′	Reverse 5′–3′
Kir6.1 (mouse)	AAGGCACCATGGAGAAGAGT	GAGAAACGCAGACGTGAATGAC
Kir6.1 (rat)	AAGGCATCACGGAGAAGAGT	GAGAAACGCAGAAGTGAATGAC
Kir6.2	AAGGCCAAGCCCAAGTTTA	CCACCACTCTACATACCATACTTCAC
SUR2	GGAGTGTGATACTGGTCCAA	ACCAGGTCTGCAGTCAGAAT
BNP	TCTCCAGAGCAATTCAAGAT	AACAACTTCAGTGCGTTACA
POSTN	CACGGCATGGTTATTCCTTCA	TCAGGACACGGTCAATGACAT
DDR2	ATCACAGCCTCAAGTCAGTGG	TTCAGGTCATCGGGTTGCAC
aSMA	GTCCCAGACATCAGGGAGTAA	TCGGATACTTCAGCGTCAGGA
IL-1b	GTACAAGGAGAACCAAGCAA	AACTCTGCAGACTCAAACTCCACTT
IL-6	AGCCAGAGTCCTTCAGAGAGATACA	GCTTATCTGTTAGGAGAGCATTGGAAATT
TGFb1	CTCCCGTGGCTTCTAGTGC	GCCTTAGTTTGGACAGGATCTG
Iba1	ATCAACAAGCAATTCCTCGATGA	CAGCATTCGCTTCAAGGACATA
beta1AR	CACTGTGGACAGCGATTCGA	ACCTTGGACTCCGAGGAGAA
RPL4	GCCAAGACTATGCGCAGGAAT	GTAGCTGCTGCTTCCAGCTT

Since myofibroblasts are the predominant cells responsible for the development of cardiac fibrosis^16^, the population of myofibroblasts was estimated by the gene expression level of alpha-smooth muscle actin (aSMA) as a specific marker of myofibroblast, compared with discoidin domain receptor 2 (DDR2) which is a marker for cardiac fibroblasts^17^. Although DDR2 expression levels in the heart remained stable with advanced age in both Kir6.1TG and WT control, the expression of aSMA in Kir6.1TG hearts stayed significantly higher levels well in advance of the elevation of periostin (Fig. 4 middle). Growth factors such as TGFβ1 and cytokines such as IL-1β and IL-6 are factors affecting fibroblast differentiation or synthesis of fibrillar collagen precursors from myoblast^16,18,19^. The expression levels of IL-1β were significantly higher than WT control while IL-6 levels are substantially higher than WT control. TGFβ1 levels are relatively higher than that of WT without significance (Fig. 4 right). Besides fibroblasts, macrophages may secrete these cytokines; however, the expression levels of Iba1 (ionized calcium binding adaptor molecule 1) as a marker for macrophage^20^ remained the similar level in all samples (Supplementary Fig. S5). These results suggest that activated fibroblasts or myofibroblasts are the major sources of these cytokines and growth factors rather than macrophages. Although the data above are staying the same throughout in each group, the sample number available at 40 weeks of age was restricted (n = 2) due to the high probability of unexpected deaths in Kir6.1TG mice. As to improve statistical data reliability, three Kir6.1TG mice at 40 weeks of age from line 111 were included in the data set of line 673 since Kaplan-Meier curves for overall survival of line 673 were comparable to those of line 111 (Fig. 1, and Supplementary Fig. S1). The data addition at 40 weeks of age from three TG mice (line 111) further supported the gene expression profiles in Kir6.1TG mouse hearts (Supplementary Fig. S6, Table 1 lower row).

## Discussion

Kir6.1TG mice, a transgenic mouse strain overexpressing vascular form Kir6.1 in cardiomyocytes, have a shorter lifespan with the median survival between 52 to 55 weeks of age. Most of the cases were reported as sudden death, beginning from late adolescent age. Catecholamine sensitivity and the cardiac fibrosis in Kir6.1TG mice indicated arrhythmogeneity as a trigger of the observed sudden deaths. Among the four different founder lines of Kir6.1TG mice, lines 673 and 634 overexpressing wild-type Kir6.1 in the heart were initially established with the goal of investigating the phenotype found in Kir6.1-deficient mice^6^. The mutant strains of Kir6.1TG mice (line 105, 111) were recently created to assess the influence of the mutation (S422L on Kir6.1) on the K_ATP_ channel activity^13^. Regardless of the mutation status, line 673 (WT) and line 111 (S422L) showed significant shortening of life expectancy at the similar degree (Fig. 1). Furthermore, the fact that line 673 and line 111 are established independently from each other strongly suggest that overexpression of Kir6.1 in cardiomyocyte is a cause of the short lifespan of the Kir6.1TG mice concerning the nature of the transgenic animals. However, transgenic mice with the α-MHC promotor are known to have multiple factors affecting pathological phenotypes, such as a combined effect of transgene integration and expression, and the promotor silencing or downregulation with age or with disease progression^21^. These factors might affect the differences in life expectancy of Kir6.1TG mice, besides the changes in ion-channel activities. *I*_KATP_ densities in the ventricular myocyte from Kir6.1TG mice are significantly smaller than WT. Among the four lines of Kir6.1TG, *I*_KATP_ densities in the group (lines 673 and 111) where the life expectancy is the shortest in Kir6.1TG lines were substantially smaller than other lines (634 and 105). Furthermore, *I*_to_ and *I*_K1_ densities in the ventricular myocyte from lines 673 and 111 were relatively larger than other lines. Further analyses on each founder line of Kir6.1TG will reveal complexed molecular mechanism affecting the electro-pathological link in the Kir6.1TG heart. Since this research mainly focuses on the cause of unexpected deaths and related changes in Kir6.1TG mice, we selected the line 673 as a representative of Kir6.1TG.

Progressive development of fibrosis and the congenital APD prolongation are pathophysiological characteristics of Kir6.1TG mice. The adult murine heart consists of myocytes and nonmyocytes including fibroblasts at similar abundance (myocytes 55%, nonmyocytes 45%)^22^. Since those fibroblasts intercalate themselves between myocytes in the heart tissue, fibrotic changes are likely to produce barriers to conduction by increasing the distance between adjacent myocytes, and decreasing myocyte-myocyte lateral coupling via gap junctions^18^. Histopathological images account for the formation of surrounding collagen fiber around myocytes (Fig. 3, TG 40 weeks, Supplementary Fig. S4 TG (line 111) 35 weeks). The aberrant ventricular conduction found in Kir6.1TG may result from this electric barrier formation through fibrosis. The prolonged APD in Kir6.1TG cardiomyocytes suggests that cardiomyocytes are under the stress condition with Ca^2+^-overload^13^, which may cause heart failure. Although BNP expression levels in Kir6.1TG mouse hearts were significantly higher than that from WT control, there was no apparent sign of lung edema derived from congestive heart failure as there were no hemosiderin-laden macrophages in the lung in Prussian blue staining. This evidence suggests that the primary cause of unexpected deaths in Kir6.1TG mice were arrhythmogenic cardiac events; however, it does not exclude the possible cases where the contraction failure of cardiomyocytes is a cause of death in Kir6.1TG mice.

Reduced cardiac K^+^-current densities such as *I*_KATP_, *I*_to_, *I*_K_, and *I*_K1_, are characteristic findings in electrophysiological changes of the Kir6.1TG mouse heart. These changes contribute to cardiac action potential prolongation, Ca^2+^-overload, and subsequent cardiac cell death. It has been reported that the transgene expression in cardiomyocyte by 𝛼-MHC promotor occasionally brings off-target effects on *I*_to_ and *I*_K1_^23^. This promotor effect could partly explain the decreased current densities of voltage-dependent K^+^-channels in Kir6.1TG cardiomyocytes. K_ATP_ channel in murine cardiomyocyte is composed of Kir6.2 and SUR2A^2,3,24,25^, and the appropriate 1:1 stoichiometry of Kir6 and SUR subunits is a requirement to form fully active K_ATP_ channel complexes on the plasma membrane^25–28^. Mismatched stoichiometry appears to be a cause of decreased *I*_KATP_ densities through the retention in the endoplasmic reticulum^29^. Kir6.1TG mouse heart where Kir6.1 is ectopically overexpressed in cardiomyocytes has reduced *I*_KATP_ density at about 40% of the WT control^13^. This phenomenon is in line with the previous reports that the heart-specific overexpression of Kir6 alone or in combination with SUR^27,30–32^ reduced K_ATP_ currents.

The characteristic finding of the Kir6.1TG heart is the progressive fibrosis (Fig. 3). The fibrosis could occur with collagen accumulation at the site of cardiomyocyte loss as a result of Ca^2+^-overload. Apparent accumulation of fibrotic staining was observed in the section from Kir6.1TG at 40 weeks, but not at nine weeks. The differentiation of fibroblast to myofibroblast is a crucial step to promote tissue repair since myofibroblasts express and secrete growth factors, cytokines, extracellular matrix, and proteases^17^. Further, myofibroblasts are not part of normal cardiac tissue and appear only following cardiac injury^18^. Marker gene analyses showed an increase of myofibroblasts in Kir6.1TG hearts from nine weeks, at the time when the pathological fibrosis had not been evident according to the collagen staining and the periostin expressions. The expression levels of the marker gene for myofibroblast (aSMA) were significantly higher than that from the WT control, although the marker for fibroblast (DDR2) remained the same as the WT control (Fig. 4). Myofibroblast is the primary source of the proinflammatory cytokine IL-1β as well as IL-6 and TNF-𝛼 in the heart^17^. Higher expression levels of IL-1β and IL-6 in Kir6.1TG mouse hearts also suggest fibroblasts differentiation. Furthermore, the fact that the expression levels of Iba1 (marker gene for macrophage) were similar between Kir6.1TG and WT control indicated that macrophages did not contribute significantly to the increase of cytokine expressions in Kir6.1TG mouse hearts. TGFβ1 stimulates the transformation of fibroblast to myofibroblast, also increases myofibroblast proliferation^18^. The substantial increase in TGFβ1 expression in Kir6.1TG mouse hearts indicates the additional involvement of unknown factor in the process of fibroblasts differentiation. Since cardiomyocytes couple directly to fibroblasts via gap junctions^18^, electrophysiological changes in myocytes also have an impact on fibroblasts. These complexed conditions may induce differentiation of fibroblasts to myofibroblasts. Electrical anisotropy in the heart refers to different specific resistances in the longitudinal and the transverse fiber direction. Since fibroblasts locate between cardiomyocytes, aligned parallel to the longitudinal axis of cardiomyocytes, fibrotic changes separate laterally neighbored cardiomyocytes^33^. Thus, conduction disturbances became the significant findings in Kir6.1TG mouse heart than contraction dysfunction.

Although the primary inducer of fibroblast differentiation to myofibroblasts in Kir6.1TG mouse hearts has not been elucidated in this research, it is highly expected that ion-channel dysregulations in ventricular myocytes are involved in the activation of the fibrosis pathway. Although ventricular fibroblasts have multiple ion channels including several potassium channels, sodium channels, and the chloride channel^34–36^, they are not electrically excitable cells, and they only pass electrical signals if externally stimulated^18^. It has been reported that ventricular fibroblasts have significant potassium conductance which may impact on fibroblast proliferation, differentiation, and function^34,35^. Therefore, once the channel function is activated or modulated by the external factor, such as K^+^ concentration in physio-pathological conditions, changes in electrical activities in cardiomyocyte may externally influence on fibroblasts and promote differentiation to myofibroblasts. Further investigations will warrant the electro-pathological substrates for cardiac remodeling.

In conclusion, the short life-expectancy of Kir6.1TG mice is the consequence of the advanced progression of cardiac fibrosis mainly initiated by the K_ATP_ channel dysregulation due to the overexpression of Kir6.1 in cardiomyocytes. Although the sample number used in this study is limited and the strict analyses using the age-matched groups were not applicable, the progressive fibrosis in Kir6.1TG mouse heart is the robust phenomenon supporting ECG changes observed. In combination with the Ca^2+^-overload due to the extended repolarization phase, advanced cardiac fibrosis played a part in the short life expectancy of the Kir6.1TG mice. This evidence will facilitate understanding of the molecular mechanism underlying cardiac K_ATP_ channelopathy^10^.

## Materials and Methods

### Kir6.1 transgenic mice

Transgenic mice (C57BL/6 background) overexpressing Kir6.1 using the α-myosin heavy chain (α-MHC) promoter linked to the Kir6.1 transgene were established previously (termed as Kir6.1TG)^13^. The transgenic mice were used as heterozygotes; WT females were bred with transgenic males at the animal facility of Chiba University. All animals received a standard mouse chow diet (NMF, Oriental Yeast, Japan) and filter-sterilized water *ad libitum* in the animal facility under controlled temperature and on a 12-h light/12-h dark cycle. All experimental procedures were approved by the animal research committee of Chiba University and performed in strict accordance with the Guidelines for Proper Conduct of Animal Experiments (Science Council of Japan, 2006) and the National Institute of Health guidelines.

Kir6.1TG strains consist of four founder lines (673, 634, 105, and 111). The first set of lines (673 and 634) express wild-type Kir6.1 from the transgene, while the following two lines (105 and 111) express Kir6.1 with mutation (S422L) from the transgene. Further description of the electrophysiological changes in Kir6.1TG heart have been reported previously^13^.

### Pharmacologic stress ECG test

Mice were anesthetized with a combination of midazolam (Astellas Pharma, Japan, 4 mg/kg), butorphanol (Meiji Seika Pharma, Japan, 5 mg/kg), and medetomidine (Nippon Zenyaku Kogyo, Japan, 0.3 mg/kg)^37^. The depth of anesthesia was maintained enough to prevent the tail pinch reflex. Surface ECG recordings were performed with standard limb leads (Lead II) during the entire period of the experiment on the temperature-controlled table. Lead signals were digitized with the sampling frequency of 4 kHz using PowerLab 6 (ADInstruments, Japan) and stored for further analysis with LabChart (ADInstruments, Japan). Following a stabilization period of about 20 minutes after the anesthesia, noradrenaline (Daiichi Sankyo, Japan) at one µg/g.BW (50 µl/25 g.BW) was intraperitoneally given repetitively in total five times at 0, 45, 90, 120, and 150 minutes, and the observation ends at 180 minutes. The maximum occurrence of aberrant ventricular waves was calculated from the 30-second sampling period between 10 to 20 minutes to infer the pharmacologic stress level induced by noradrenaline.

### Tissue specimen

The hearts taken as en bloc with the lung were fixed in 10% formalin immediately after the removal of the ventricular apex for RNA extraction. The 2-µm-thick sections of paraffin-embedded tissues were used for hematoxylin and eosin stain, Masson’s trichrome stain, and EVG stain. The lung sections were further analyzed with Prussian blue stain.

### Measurement of gene expressions

Gene expression levels were analyzed by semi-quantitative real-time PCR. Mouse heart was quickly removed after sacrifice; ventricular apex tissue was sampled, stored in RNAlater Stabilization Solution (AM7024 Ambion, USA) at −80 °C until use. Total RNA was extracted with RNAiso PLUS (9108 Takara Bio, Japan), followed by the further purification with RNeasy mini-kit (74104 Qiagen, USA) according to the manufacturer’s instruction. Two μg of total RNA was then used to synthesize first strand cDNA with a SuperScript VILO cDNA Synthesis Kit (11754050 Invitrogen, USA). mRNA levels were quantified by real-time PCR with SYBR green dye (QPS-201 Thunderbird Sybr qPCR Mix Toyobo, Japan) with the specific primer sets^13,38,39^ shown in Table 2, and normalized to ribosomal protein L4 (RPL4) mRNA^40^.

### Data Analysis

All data were expressed as mean ± standard error (SE) of the mean unless otherwise mentioned. Differences between groups were assessed by one-way analysis of variance (ANOVA) with post-hoc Bonferroni HSD test, or Kruskal-Wallis test with Bonferroni HSD test where applicable. P values of <0.05 were considered significant unless otherwise stated. All statistical analyses were performed with EZR (Ver.1.3.5, Saitama Medical Center, Jichi Medical University, Japan)^41^, which is a graphical user interface for R (Ver.3.4.1, The R Foundation for Statistical Computing, Austria).

### Data Availability Statement

Kir6.1 transgenic mouse strains (Kir6.1TG line 634, 673, 105, and 111) used in this study are available through RIKEN BioResource Center (RBRC10018-21).

## Electronic supplementary material

Supplementary Information
