# Supplementary material for: Ectopic overexpression of Kir6.1 in the mouse heart impacts on the life expectancy
Source: Sci Rep. 2018 Aug 6;8:11723. doi: 10.1038/s41598-018-30175-5 (PMC6078942; doi:10.1038/s41598-018-30175-5)
Supplement: Supplementary file 1 — Supplementary Information [file 41598_2018_30175_MOESM1_ESM.pdf]

Supplementary Information

Ectopic overexpression of Kir6.1 in the mouse heart impacts on the life expectancy

Yasuhiro Watanabe[1], Takashi Kishimoto[2], Takashi Miki[3], Susumu Seino[4], Haruaki Nakaya[1], and Akio Matsumoto[1]

Department of Pharmacology[1], Department of Molecular Pathology[2], Department of Medical Physiology[3], Graduate school of Medicine, Chiba University, Chiba, Japan

Division of Molecular and Metabolic Medicine[4], Kobe University Graduate School of Medicine, Kobe, Japan

Supplementary Figure S1, S2, S3, S4, S5, S6

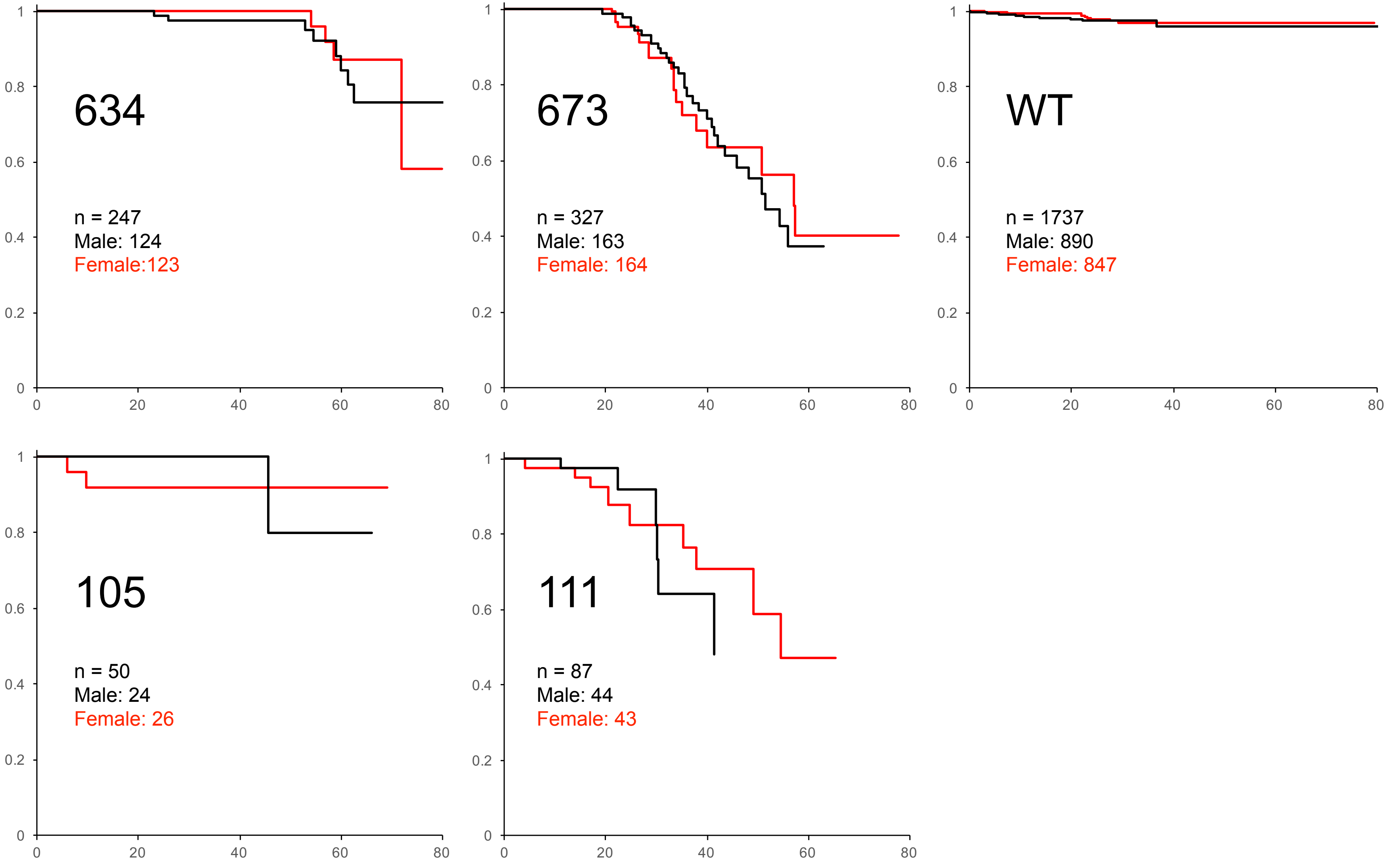

Supplementary Figure S1: Kaplan-Meier curves for overall survival among each mouse strains.

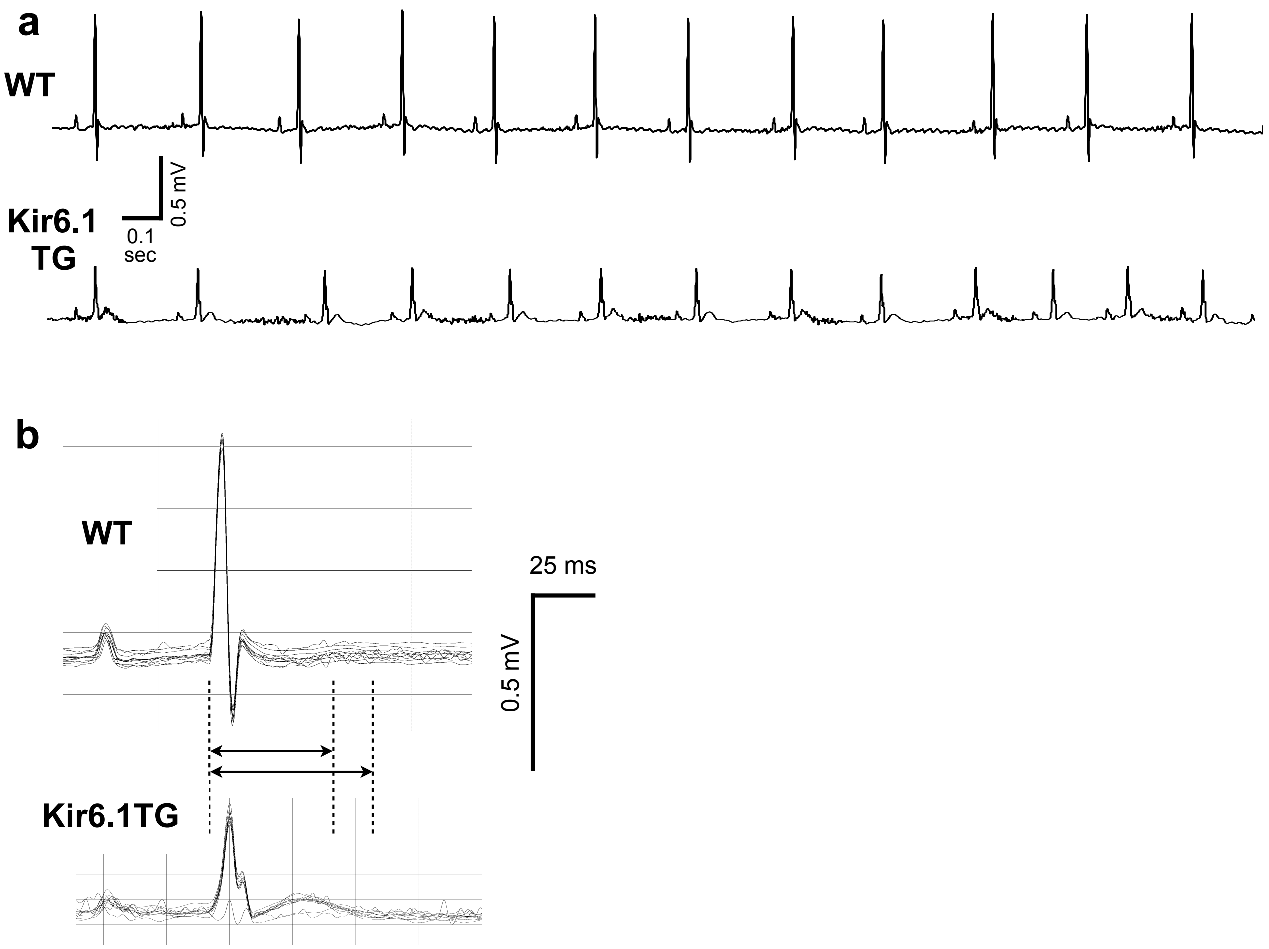

Supplementary Figure S2: Surface ECG traces from WT and Kir6.1TG (Line 673) mice.
a: ECG recordings (lead-II) from anesthetized mice just before an administration of noradrenaline. Heart rate was approximately WT:248, Kir6.1TG:227/min, regular sinus rhythm. **b**: The averaged ECG traces (dark line) from 10 consecutive beats (lines in light gray). Extension of QT interval in the Kir6.1TG mouse was shown with dotted lines.

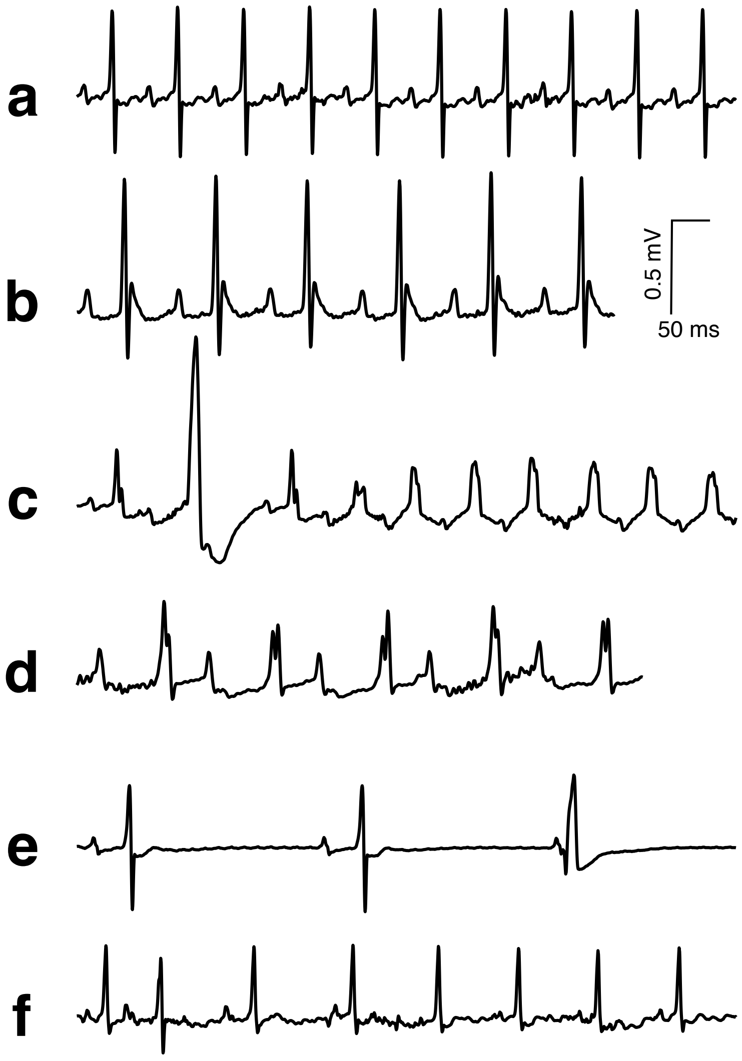

Supplementary Figure S3: Atypical ECG patterns observed in the stress-ECG recordings. Surface ECG (lead-II) was recorded from the anesthetized mouse. An administration of noradrenaline (1 mg/kg.BW i.p.) induced tachycardia (**a** :Kir6.1TG (Line 673), **b** :WT), and aberrant conduction mainly in Kir6.1TG (Line 673) mice (**c-f**). Many of the atypical ECG patterns suggested ventricular conduction delay (**c, d**), but there were some patterns indicating supraventricular abnormal rhythm or conduction (**e, f**).

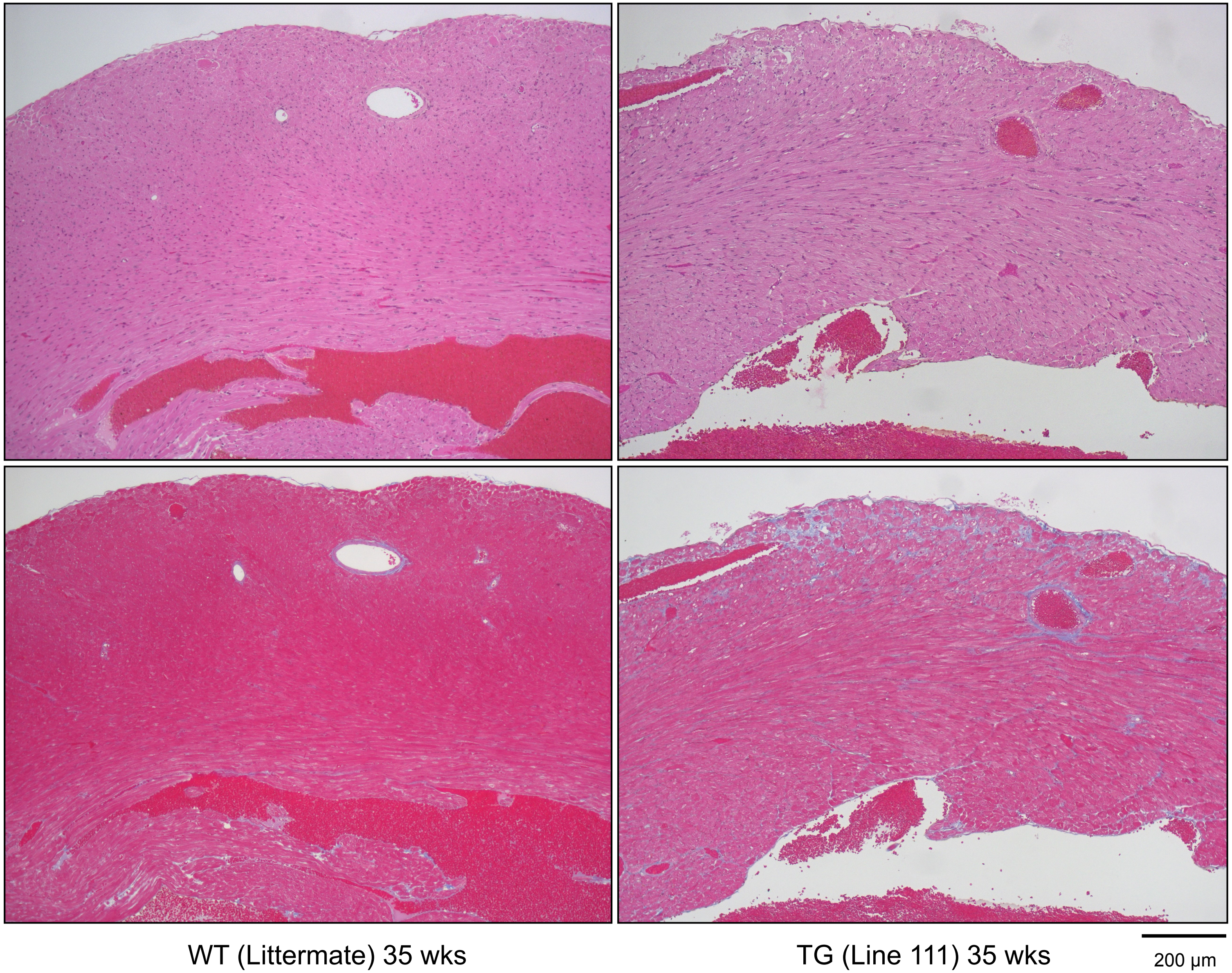

Supplementary Figure S4: Fibrotic changes in Kir6.1TG mouse heart (Line 111). Representative Hematoxylin and Eosin staining (Upper panels) and Masson Trichrome staining (Lower panels) of the anterior wall of the left ventricle section from Kir6.1TG (Line 111) mice and WT littermate (n = 2 mice per group, age at 35-wks). Kir6.1TG heart increases in fibrosis (Blue) compared to the age-matched WT littermate (35 wks). Scale bar: 200 µm, original magnification x100.

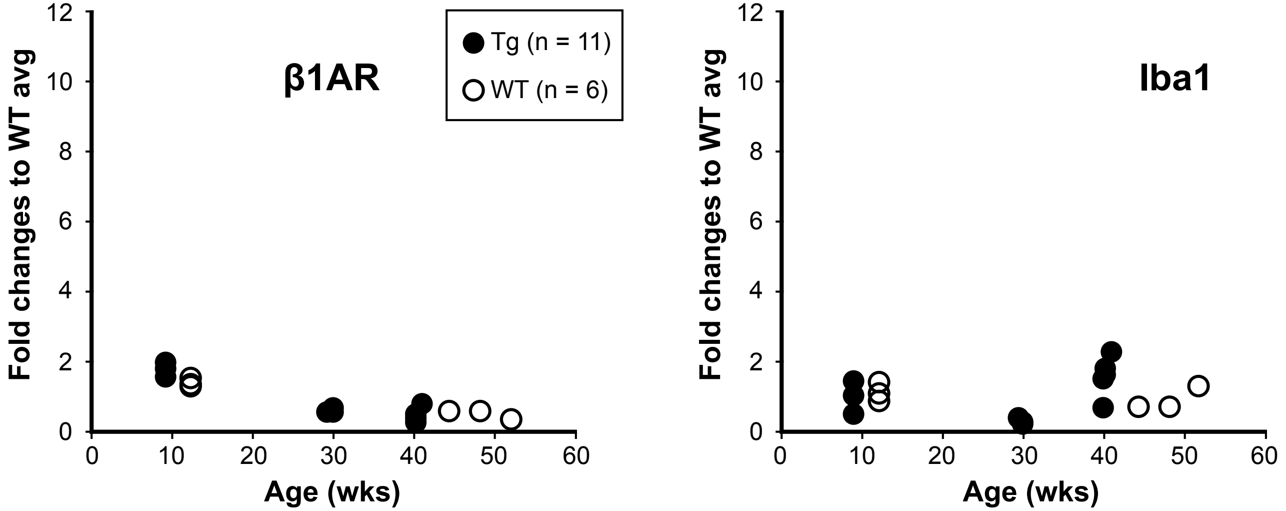

Supplementary Figure S5: Gene expression analyses in the apex tissue by semi-quantitative real-time PCR. cDNA was synthesized from the total RNA purified from the apex of the left ventricle. Kir6.1TG mice (Line 673) were sub-grouped according to the age of weeks (9, 30, 40, respectively), and compared with WT (average 48.2 wks and 12 wks). qPCR values were first normalized to RPL4, then calculated fold changes to the average of WT. Each circle indicates the value of each mouse (WT: n = 6, TG 9 wks: n = 3, 30 wks: n = 3, 40 wks: n = 5 (including n = 2 from Line 673 and n = 3 from Line 111). β1AR: β1-adrenergic receptor, Iba1: ionized calcium binding adaptor molecule 1.

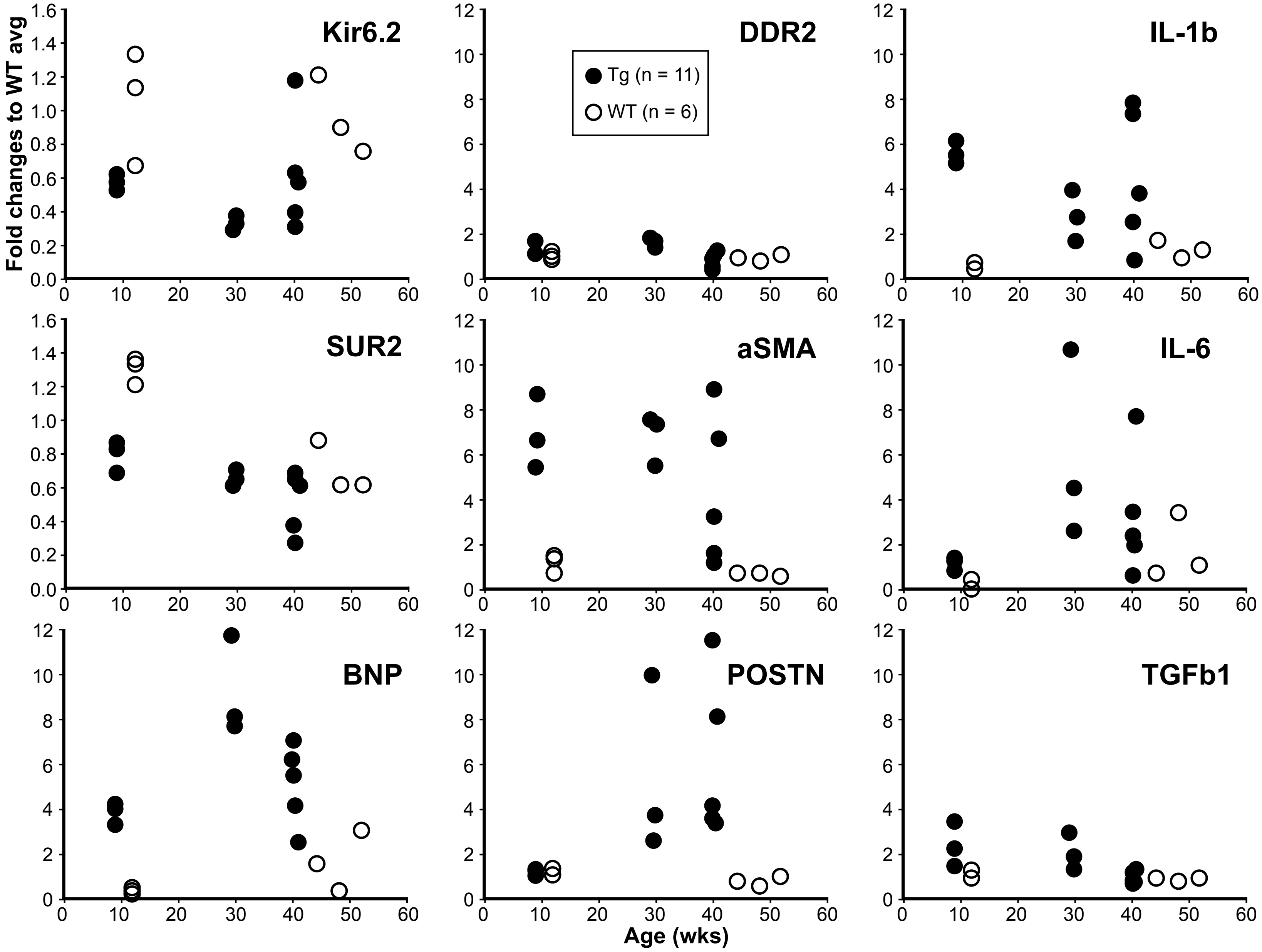

Supplementary Figure S6: Gene expression analyses in the apex tissue by semi-quantitative real-time PCR. cDNA was synthesized from the total RNA purified from the apex of the left ventricle. These figures are almost identical to Figure 4, with an addition of data from three mice (Line 111) in the group of 40-wks. Mice were sub-grouped according to the age of weeks (9, 30, 40, respectively), and compared with WT (average 48.2 wks and 12 wks). qPCR values were first normalized to RPL4, then calculated fold changes to the average of WT. Each circle indicates the value of each mouse (WT: n = 6, TG 9 wks: n = 3 (Line 673), 30 wks: n = 3 (Line 673), 40 wks: n = 5 (including n = 2 from Line 673, and n = 3 from Line 111). Kir6.1r: rat Kir6.1 (transgene), Kir6.2: mouse Kir6.2, SUR2: sulfonylurea receptor type 2, BNP: B-type natriuretic peptide, POSTN: periostin, DDR2: discoidin domain receptor 2, aSMA: alpha-smooth muscle actin, IL-1b: interleukine-1β, IL-6: interleukine-6, TGFb1: transforming growth factor β1.
